# A Crisscross-Strategy-Boosted Water Flow Optimizer for Global Optimization and Oil Reservoir Production

**DOI:** 10.3390/biomimetics9010020

**Published:** 2024-01-02

**Authors:** Zongzheng Zhao, Shunshe Luo

**Affiliations:** School of Geosciences, Yangtze University, Wuhan 430100, China; zhaozz_ytu@hotmail.com

**Keywords:** water flow optimizer, production optimization, global optimization, crisscross mechanism, metaheuristic algorithms, bionic algorithm

## Abstract

The growing intricacies in engineering, energy, and geology pose substantial challenges for decision makers, demanding efficient solutions for real-world production. The water flow optimizer (WFO) is an advanced metaheuristic algorithm proposed in 2021, but it still faces the challenge of falling into local optima. In order to adapt WFO more effectively to specific domains and address optimization problems more efficiently, this paper introduces an enhanced water flow optimizer (CCWFO) designed to enhance the convergence speed and accuracy of the algorithm by integrating a cross-search strategy. Comparative experiments, conducted on the CEC2017 benchmarks, illustrate the superior global optimization capability of CCWFO compared to other metaheuristic algorithms. The application of CCWFO to the production optimization of a three-channel reservoir model is explored, with a specific focus on a comparative analysis against several classical evolutionary algorithms. The experimental findings reveal that CCWFO achieves a higher net present value (NPV) within the same limited number of evaluations, establishing itself as a compelling alternative for reservoir production optimization.

## 1. Introduction

In the fields of engineering, energy production, and various industries, decision makers face increasingly complex challenges [1,2,3]. The need for optimization strategies has grown alongside the increasing complexity of these problems. Optimized solutions are essential due to the intricate interplay of factors such as resource scarcity, economic constraints, and technological advancements [4]. Whether in the intricate task of global optimization [5] or the nuanced field of oil reservoir production [6], the imperative for streamlined and efficient solutions becomes paramount.

In order to tackle these challenges, optimization methods play a crucial role in problem-solving approaches. These methods can be broadly divided into deterministic methodologies and metaheuristic algorithms [7]. Deterministic methods, characterized by their structured and precise approach, offer advantages in terms of convergence and reliability; examples include conjugate gradient methods [8], linear programming [9], interior point methods [10], simplex methods [11], etc. However, they usually require the problem to be convex, differentiable, continuous, etc., and it is difficult to solve complex high-dimensional problems. On the other hand, metaheuristic algorithms have almost no requirements on the properties of the problem and bring a new perspective to optimization due to their adaptive and stochastic properties. Nevertheless, their extensive computational requirements and a lack of guaranteed convergence remain notable challenges.

Among metaheuristic algorithms, two prominent classifications are Swarm Intelligence Algorithms (SIs) and evolutionary algorithms (EAs). SIs and EAs have a similar structure, where a set of solutions is first randomly initialized, after which new offspring are generated using a set update strategy, and finally the solutions of the new generation are selected using a specific selection strategy. This process is repeated until the maximum number of iterations is reached. SIs are mainly inspired by the aggregation behaviors of biological population intelligence, among which the classical ones include the Particle Swarm Optimizer (PSO) [12], Ant Colony Optimizer (ACO) [13], and emerging algorithms in recent years, including the Harris Hawk Optimizer (HHO) [14], Grey Wolf Optimizer (GWO) [15], artificial bee colony (ABC) optimization algorithm [16], Hunger Games Search (HGS) [17], Slime Mushroom Algorithm (SMA) [18], Lunger–Kuta Optimization Algorithm (RUN) [19], Vector Weighted Average Algorithm (INFO) [20], etc. EAs mainly simulate the evolutionary process of natural selection [12] and survival of the fittest. They typically involve three operators: crossover, mutation, and selection. Classical EAs include a Genetic Algorithm (GA) [21], Genetic Programming (GP) [22], Spherical Evolution (SE) [23], differential evolutionary (DE) [24].

The intricacies of problem solving in diverse industrial landscapes are further underscored by the No Free Lunch Theorem (NFL) [25], which postulates the absence of a universally superior optimization algorithm. In essence, this theorem asserts that the performance of any given algorithm is contingent upon the specific characteristics of the problem at hand. Consequently, the adoption of tailored algorithms to address specific industrial challenges becomes imperative. Recognizing the inherent limitations and idiosyncrasies of existing algorithms, the impetus for algorithmic improvement gains prominence in navigating the intricate terrain of optimization.

In the field of oil reservoir development, creating an effective production scheme is essential for efficient recovery and sustained production. Optimizing the injection and production processes in oil reservoirs involves considering various dynamic factors, such as reservoir heterogeneity, fluid properties, and operational constraints. Striking an optimal balance in injection rates and production strategies is vital for maximizing hydrocarbon recovery and minimizing operational costs [26]. The inherent complexities of this domain call for innovative approaches to address the multifaceted challenges posed by the dynamic nature of oil reservoirs.

Numerous scholars have made efforts to improve the optimization of petroleum injection and production. Foroud et al. [27] applied eight different optimization algorithms to the optimization of oil and gas production in the Bruges field. The results show that the Guided Pattern Search (GPS) is the most effective and gives the most NPV in the least number of evaluations. Ying et al. [28] proposed a multi-fidelity migrated differential evolutionary algorithm (MTDE), which utilizes the results of different fidelity levels to exchange and migrate information, accelerating the convergence of the algorithm and improving the quality of the optimal knots. The proposed method is validated on an egg model and two real field case models for production optimization, and the results show that the MTDE has a faster convergence rate and a higher quality well control strategy than the single-fidelity and greedy multi-fidelity methods. Zhang et al. [29] proposed a two-model differential evolutionary algorithm (CSDE) for constrained water drive optimization by constructing the boundaries of the feasible domain shown by a support vector machine, after which the objective function is approximated using a Radial Basis Function (RBF). The proposed algorithm is applied to a case study, and the results show that CSDE can effectively handle the constraints, and higher NPV can be obtained compared to other single-model algorithms. Desbordes et al. [30] proposed a migration learning-based optimization framework for solving dynamic production optimization problems. The developed framework is integrated into three well-known evolutionary algorithms, the Non-dominated Sequential Genetic Algorithm (NSGA-II), the Multi-Objective Particle Swarm (MOPSO) and the Decomposition-based Multi-Objective Evolutionary Algorithm (MODE), and PSO. The proposed method was tested on 12 benchmark functions and a real amenity, and in comparison, with their original method, the method effectively reduces the number of calls to the numerical simulator and achieves better NPV. There are many similar studies, but they usually focus on the construction of the agent model and neglect the choice of the optimization framework (DE or PSO is always chosen as the optimization framework). However, an optimizer adapted to a specific task is crucial for achieving better optimization results.

The water flow optimizer (WFO) is a nature-inspired evolutionary algorithm that was introduced in a prestigious journal in 2021 [31]. Its convergence has been rigorously established through limit theory, and it has demonstrated successful applications in the optimization of spacecraft trajectories. Chen et al. [32] proposed an enhanced water flow optimizer and a refined maximally similar path localization algorithm (IWFO-IMSP) for precise localization of wireless sensor networks. These advancements significantly enhance the convergence speed and capability of WFO by integrating strategies like Halton sequences and Cauchy variants. Notably, the proposed IWFO-IMSP algorithm showcases considerably superior localization accuracy when compared to four traditional methods. Furthermore, Xue et al. [33] proposed an enhanced WFO that adaptively tunes hyperparameters of a theory-guided neural network. The quality of the initial population is enhanced through the application of an adversarial learning technique during the initial stage of WFO, while the diversity of the population is improved by introducing a nonlinear convergence factor to the laminar flow operator. This framework exhibits superior performance in solving stochastic partial differential equations. In another study, Fagner et al. [34] proposed a binary variation of WFO that exhibits superior results compared to other classical dimensionality reduction methods. These studies collectively indicate that, despite the superior optimization performance of WFO, further enhancements are necessary to tailor it to specific problem domains.

In this paper, an enhanced WFO algorithm called CCWFO is proposed. It is used to significantly enhance the global optimization capability of the original algorithm and makes it effectively applicable to oil production optimization, by introducing the CC mechanism to enhance the information interaction among the individuals in the population, to enrich the diversity of the population.

The main contributions of this paper are as follows:

- An enhanced WFO algorithm is proposed by introducing the CC mechanism.
- The performance of the CCWFO algorithm is verified in detail, through comparison experiments with 10 other conventional and state-of-the-art optimization algorithms on the CEC2017 benchmark function, and the experimental results obtained are additionally subjected to W and F tests.
- The proposed algorithm is used to solve production optimization problems based on three-channel reservoirs.

The structure of the paper is as follows: Section 1 introduces the background of this research and motivation, briefly performs a literature review, and concludes with a summary of the main contributions of this paper. Section 2 briefly describes the original WFO algorithm. Section 3 describes the CC mechanism and presents the proposed CCWFO algorithm in detail. Section 4 describes the flow, results, and analysis of the global optimization experiments. Section 5 presents an example of the application of CCWFO in a three-channel reservoir. Section 6 summarizes the whole paper.

## 2. Overview of the Original WFO

WFO, a Swarm Intelligence (SI) Algorithm proposed by Prof. Kaiping Luo in 2021 [31], draws inspiration from the two distinctive types of water flows found in nature: laminar and turbulent. In nature, water flows from high to low, which is similar to the process of searching for a solution in an optimization problem. In the WFO algorithm, the water particles are considered as the solution, the positions of the water particles are considered as the values of the solution, and the potential energy of the water is considered as the fitness value of the objective function. The algorithm simulates the behavior of laminar and turbulent flows in the water flow process through mathematical modelling and finds the optimal solution through continuous iteration. The mathematical description of laminar and turbulent flow is as follows:

**1. Laminar Operator**: In laminar flow, all particles move parallel to each other in the same direction, but their speed varies due to the surroundings. The rule of motion is denoted using Equation (1).

(1) $$y_{i}\left(t\right)=x_{i}\left(t\right)+s*d\;\;\;\;\;\;\;\;\forall i\in \left\{1,2,\ldots ,m\right\}$$

where t is the current iteration number, m is the population size, $x_{i}\left(t\right)$ is the position of the i^th^ particles at the t^th^ iteration, $y_{i}\left(t\right)$ is the possible movement position of the t^th^ individual at the t^th^ iteration, s is a random number between 0 to 1, and the d vector represents the common direction of movement of all the individuals at the current iteration; d is defined as shown in Equation (2).

(2) $$d=x_{best}-x_{k}\left(t\right),\;\;\;x_{best}\neq \;x_{k}\left(t\right)$$

where $x_{best}$ represents the optimal solution obtained by the current iteration of the population and $x_{k}\left(t\right)$ represents a randomly selected particle in the population.

In the laminar flow operator, individuals in the population use a regular parallel unidirectional search, where the same direction vector d ensures that the search is unidirectional, and the randomness of s ensures that different individuals have different move steps.

**2. Turbulen Operator:** In turbulence, water particles are affected by other obstacles and show irregular rotational movements. The possible moving position $y_{i}$ is generated by the random dimension of the i^th^ individual through Equation (3).

(3) $$y_{i}=\{\begin{array}{ll} x_{i}^{j1}\left(t\right)+\left|x_{i}^{j1}\left(t\right)-x_{k}^{j1}\left(t\right)\right|*\theta *cos\left(\theta \right), & \;if\;r<p_{e}\\ \left(ub^{j1}-lb^{j1}\right)*\frac{x_{k}^{j2}\left(t\right)-lb^{j2}}{ub^{j2}-lb^{j2}}+lb^{j1}, & \;otherwise\; \end{array}$$

The upper part of Equation (3) denotes the vortex transformation of water particles in the same layer and the lower part of Equation (3) denotes general cross-layer movement of particles. Where j1 denotes a dimension randomly selected from the particle, j2 denotes a dimension different from j1 randomly selected from the particle, and $x_{k}^{j1}\left(t\right)$ denotes the value of the *j*1th dimension of the k^th^ particle at the t^th^ iteration. θ is a random number in the range −π to π, $ub^{j1}$ and $lb^{j1}$ denote the upper and lower bounds of the selected dimension, respectively, r is a random number in the range 0 to 1, and $p_{e}\in \left(0,1\right)$ is a control parameter called the vortex probability.

**Figure 1:**
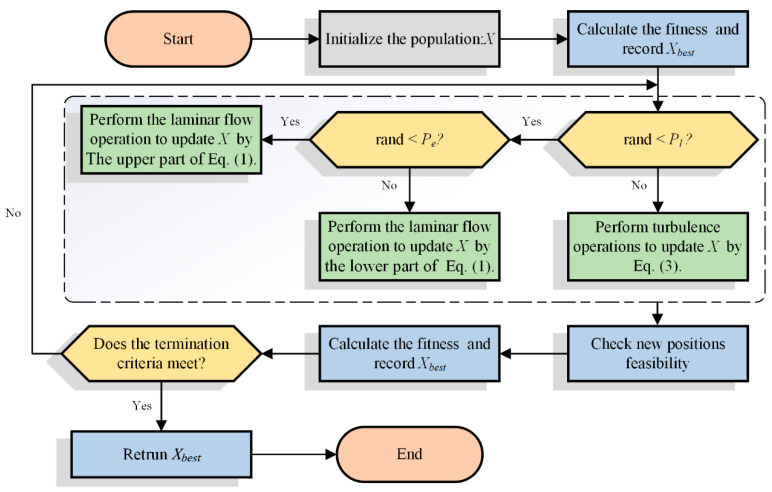
Flowchart of WFO.

During the iterations of the algorithm run, the algorithm performs a stochastic simulation of the two behaviors, laminar and turbulent, and their respective run probabilities are controlled by the parameter $p_{l}$. All generated solutions are evaluated and the new particles generated by each iteration update are compared with the old particles and the particles with better fitness values are retained. The iteration is repeated until the termination condition is met; the flowchart of the algorithm for WFO is shown in Figure 1.

## 3. Proposed CCWFO

### 3.1. Crisscross Strategy

The crossover mechanism draws inspiration from Meng’s crossover optimizer (CSO) [35], which was proposed in 2014. It incorporates two operations, namely a horizontal crossover search (HCS) and vertical crossover search (VCS), representing the exchange of horizontal and vertical information between particles, respectively. Essentially, the core concept of the crossover strategy involves generating new particles by exchanging information between randomly selected particles or dimensions. The fittest particles are retained and added to the population. This crossover mechanism exhibits a strong global search capability. Shan et al. [36] enhanced the CSA by integrating the crossover strategy and the combined mutation strategy, where the crossover strategy effectively facilitated the population in escaping local optima. Hu et al. [37] introduced the crossover strategy into the SCA algorithm and experimentally demonstrated that it accelerated the global convergence of the population, improved population diversity, and aided particles in escaping local optima.

In this study, we introduce the crossover mechanism into WFO to enhance its single search mode and enrich population diversity. This exchange of information between particles accelerates the algorithm’s convergence and improves its ability to escape local optima. HCS and VCS are described as follows.

#### 3.1.1. Horizontal Crossover Search

HCS refers to the crossover operation of the dimensions of two randomly selected particles, which can make more use of the population information, refine the search process, and improve the algorithm’s global exploration capability. HCS operation is defined using Equations (4) and (5).

(4) $$HCS_{i}^{j}=r_{1}\times x_{i}^{j}+\left(1-r_{1}\right)\times x_{k}^{j}+c_{1}\times \left(x_{i}^{j}-x_{k}^{j}\right)$$

(5) $$HCS_{k}^{j}=r_{2}\times x_{k}^{j}+\left(1-r_{2}\right)\times x_{i}^{j}+c_{2}\times \left(x_{k}^{j}-x_{i}^{j}\right)$$

where $r_{1}$ and $r_{2}$ are random numbers within the range [0, 1], $c_{1}$ and $c_{2}$ are random numbers within the range [−1, 1], $x_{i}^{j}$ is the value of the j^th^ dimension of the i^th^ particle of population X, and $x_{k}^{j}$ is the value of the j^th^ dimension of the k^th^ particle of population X. $HCS_{i}^{j}$ and $HCS_{k}^{j}$ are the new offspring of the two particles generated by the HCS operation. After the HCS operation, the new offspring will compete with the parental particles, retaining the particles with better fitness. The pseudo-code for HCS is shown in Algorithm 1.
**Algorithm 1** Horizontal crossover searchBhc = randperm (n) **For** ii=1:n/2   i  = Bhc (2ii−1)   k  = Bhc (2ii)   **For** j=1:dim    Generate four random number $r_{1}$, $r_{2}\in$(0,1), $c_{1}$, $c_{2}\in$(−1,1)    Generate $HCS_{i}^{j}\;$ and $HCS_{k}^{j}\;$by Equations (4) and (5)  **End** For **End**
For **For** i=1:n  **IF**
 fitness ($HCS_{i}$) <  fitness ($X_{i}$)                  X(i)←HCS(i)  **End** IF **End** For **End**

#### 3.1.2. Vertical Crossover Search

The VCS operation is performed for each particle by randomly selecting a set of two pairs of dimensions for crossover to obtain a new particle. Similarly, after the VCS operation, the offspring particles will compete with the parental particles and ultimately retain the better ones. VCS operation is defined using Equation (6).

(6) $$VCS_{i}^{j}=r_{3}\times x_{i}^{j1}+\left(1-r_{3}\right)\times x_{i}^{j2}$$

where $r_{3}$ is a random number within the range [0, 1], $x_{i}^{j1}$ and $x_{i}^{j2}$ represent the values of the two dimensions randomly selected by the i^th^ individual, respectively, and $VCS_{i}^{j}$ represents the value of the j^th^ dimension generated from two random dimensions of the i^th^ particle. The pseudo-code for VCS is shown in Algorithm 2.
**Algorithm 2** Vertical crossover searchBvc = randperm (dim) Generate a random number p∈(0,1) **For** j=1:dim/2    **IF**
p < 0.6    j1 = Bvc (2j−1)      j2 = Bvc (2j)   **For**  i=1:n        Generate a random number $r_{3}\in$(0,1)        Generate $VCS_{i}^{j}$ by Equation (6)   **End**
For   **End**
IF **End**
For **For** i=1:n    **IF**
 fitness (VCS(i)) < fitness (X(i))       X(i)←VCS(i)   **End**
IF **End**
For **End**

### 3.2. The Proposed CCWFO

**Figure 2:**
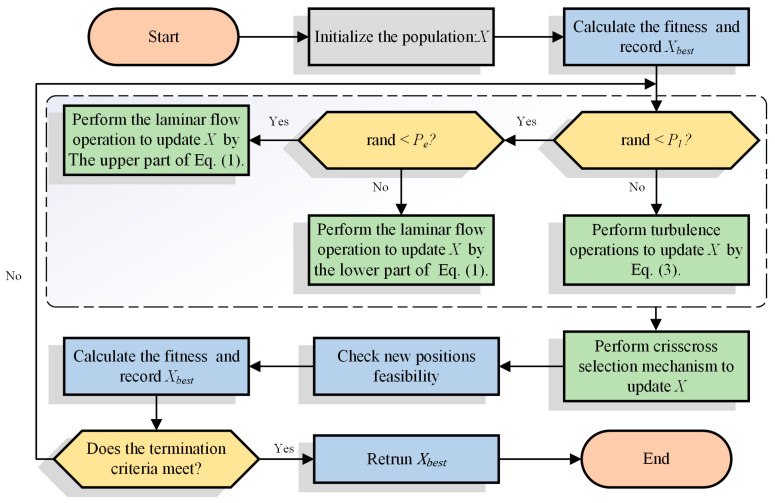
Flowchart of CCWFO.

In this subsection, the specific workflow of CCWFO is described. Firstly, CCWFO initializes the initial population of the algorithm and the required parameters, after which the algorithm sequentially updates the particles in the population according to the laminar and turbulent operations of the original WFO. At the end of the update strategy for laminar and turbulent flow, the algorithm will execute the CC strategy to enhance the information exchange between the population particles through HCS and VCS operations to explore the search space in more detail. Finally, this process will be iterated until the termination condition of the algorithm is reached and the current globally optimal particle is finally returned. The flowchart of the algorithm is shown in Figure 2.

The pseudo-code of CCWFO is given in Algorithm 3.
**Algorithm 3** Pseudo-code of CCWFOSet parameters: The maximum iteration number T, the problem dimension dim, and the population size N Initialize population X t = 1
**For** i=1:N   Evaluate the fitness value of $x_{i}$   Find the global min $x_{best}$ **End** For **While (**t≤T)    **IF** $rand<p_{l}\;$      **For** i=1:N                   /* Laminar flow */        **For** j=1:dim           Generate $y_{i}\;$by Equations (1) and (2)                  **End**
For      Evaluate the fitness value of $y_{i}$      Update $x_{i}$, $X_{best}$      **End**
For     **Else**
     **For** i=1:N                 /* Turbulent flow */
        **For** j=1:dim           Generate $y_{i}\;$by Equation (3)         **End**
For      Evaluate the fitness value of $y_{i}$      Update $x_{i}$, $X_{best}$      **End**
For   **End IF**   **For** i=1:N                         /*CC*/     Perform Horizontal crossover search to update $x_{i}$      Perform Vertical crossover search to update $x_{i}$      Update $X_{best}$   **End**
For   t=t+1;
**End While**
**Return** $X_{best}$ **End**


The time complexity of the CCWFO algorithm can be succinctly deconstructed into a composite of four stages: population initialization, laminar and vortex operations, and the CC strategy. The paramount parameters that exert a significant influence on the time complexity encompass the dimensionality (dim), the total iterations (T), and the population size (N). Consequently, the time complexity of CCWFO, denoted as O(CCWOF), may be delineated as follows: O(CCWOF) = O(initialization) + O(WFO) + O(CC) ≈ O(n × dim) + O(T × n × dim) + O(T × dim) ≈ O(T × dim × N).

## 4. Global Optimization Experimental Results and Analysis

This section presents a comprehensive and rigorous evaluation of the proposed CCWFO from a global optimization perspective using different types of benchmark functions. All the experiments were conducted fairly on benchmarks that comply with industry accepted standards. The experiments were conducted on a computer equipped with Intel Xeon Silver 4110 CPU and 128 GB RAM with Windows 10 as the operating system, and all algorithms were coded on MATLAB 2020B. The same parameter settings for all algorithms are as shown in Table 1.

### 4.1. Benchmark Function

In this subsection, we succinctly introduce the 29 benchmark functions employed in The IEEE Congress on Evolutionary Computation (CEC) [38]. It is worth noting that function F2 has been officially expunged from consideration due to its inherent propensity for inducing instability. These aforementioned 29 functions have been systematically categorized into four distinct types, namely unimodal, multimodal, hybrid, and composition. This meticulous categorization serves the noble purpose of guaranteeing a comprehensive and equitable assessment of the test functions, thereby upholding the rigorous standards of evaluation. A brief description of CEC 2017 is given in Table 2.

### 4.2. Performance Comparison with Other Algorithms

In this subsection, the comparative results of CCWFO and 10 other algorithms on the CEC 2017 benchmark are presented. These 10 algorithms encompass a mix of classical metaheuristics and advanced algorithms that have emerged in recent years. Specifically, the algorithms considered are WFO [31], SMA [18], WOA [39], PSO [12], GWO [15], MFO [40], BMWOA [41], RCBA [42], SCADE [43], and OBSCA [44]. The hyperparameters associated with each algorithm are presented in Table 3.

The experimental results obtained by CCWFO and other algorithms on each benchmark function of CEC2017 are given in Table 4, where ‘Rank’ denotes the Friedman test rank of the algorithm, ‘AVG’ denotes the average of the rankings obtained by the algorithm on each function of CEC2017, and ‘/−/=’ denotes that CCWFO is better than, equal to, or superior to other algorithms.

Table 4 shows that the average ranking of CCWFO on the benchmark function is 1.3793, which is ranked first among all competitors, indicating that CCWFO has a significant advantage over other algorithms. CCWFO obtained the global optimum in all 30 runs on F3 and F6 and was close to the global optimum on F5, F7, F8, F9, F11, F14, F15, F18, F19, and F20. This shows the stability of the algorithm’s optimization ability to obtain stable optimization results. Among the compared algorithms, WFO performs closest to CCWFO, but also performs worse than the proposed algorithm on 14 functions.

Table 5 reinforces the points obtained in Table 4. In the Wilcoxon signed-rank test, a *p* < 0.05 means that the hypothesis can be rejected, meaning that the algorithm is significantly different compared to the comparison algorithms. In Table 5, we can see that, mostly, *p* < 0.05 on most of the functions, which provides strong evidence that CCWFO significantly outperforms the other algorithms on the benchmarks.

**Figure 3:**
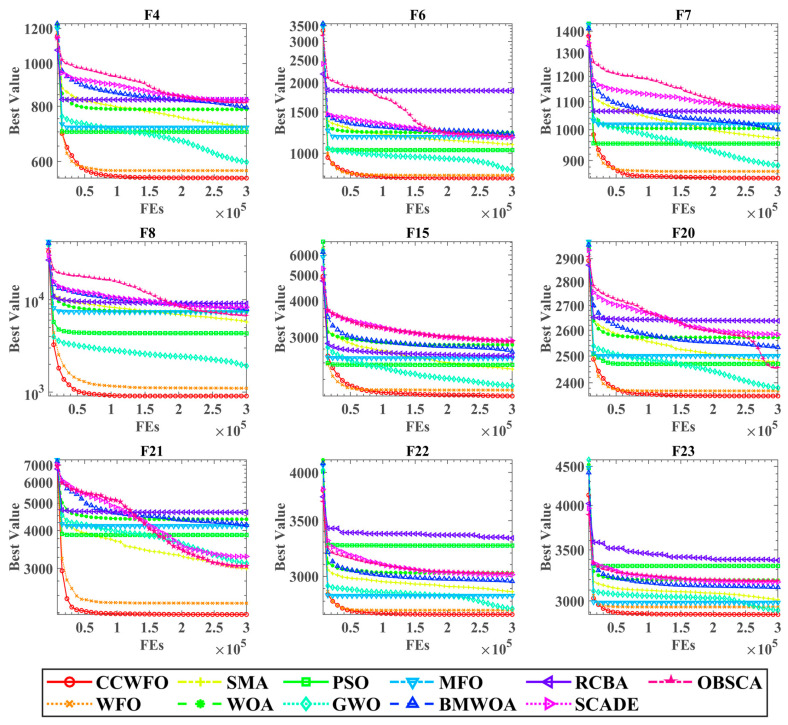
Convergence curves of CCWFO on benchmarks with other algorithms.

Figure 3 displays the convergence curves of all the algorithms on selected functions. The horizontal axis represents the number of evaluations conducted by the algorithms, while the vertical axis represents the current best fitness value achieved by the algorithms. The legend, located at the bottom of Figure 3, provides information about the different algorithms. Notably, the red lines consistently remain below the other colored lines across all function types. This observation indicates that CCWFO successfully escapes local optima and discovers superior solutions compared to the other algorithms. In conclusion, CC effectively improves the search performance of WFO and has a significant advantage over other algorithms on the benchmark.

## 5. Application to Oilfield Production

The objective of reservoir production optimization is to identify the optimal solution for each well in order to maximize NPV, and a combinatorial explosion of solution designations occurs due to the larger number of wells and production cycles leading to larger dimensions of optimization variables. Therefore, the problem can be regarded as a typical NP-hard problem, which creates conditions for the introduction of evolutionary algorithms. In this section, based on the reservoir numerical simulation software Eclipse 2010.1, CCWFO is applied to a three-channel reservoir model, and the performance of the method is compared with several classical evolutionary algorithms.

Disregard the nonlinear constraints in oilfield production and take the net present value (NPV) as the objective function to be optimized, and the specific description of NPV is shown in Equation (7).

(7) $$NPV\left(x,z\right)=\overset{n}{\underset{t=1}{\sum }}\Delta t\frac{Q_{o,t}\cdot r_{o}-Q_{w,t}\cdot r_{w}-Q_{i,t}\cdot r_{i}}{{\left(1+b\right)}^{p_{t}}}$$

where x is the set of variables to be optimized; in this experiment, the variables are the injection and recovery rates of each well. z is the state parameter of the model, which is used to describe the construction of the numerical reservoir model, n denotes the total simulation time, and $Q_{o,t}$, $Q_{w,t}$, and $Q_{i,t}$ are the oil production rate, water production rate, and water injection rate, respectively, at time step t. $r_{o}$ is the oil revenue, $r_{w}$ and $r_{i}$ are the cost of treating and injecting the water, respectively, b is the average annual interest rate, and $p_{t}\;$is the number of years elapsed.

### 5.1. Three-Channel Model

**Figure 4:**
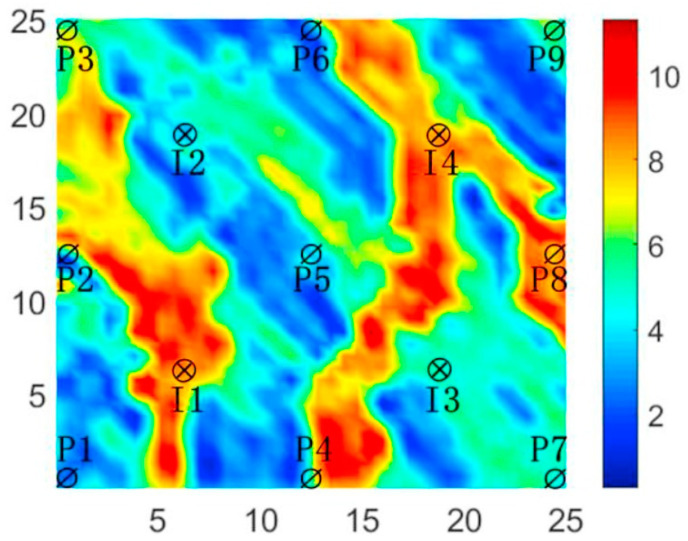
Log-permeability distribution of the three-channel model.

The three-channel reservoir model is a typical non-homogeneous two-dimensional reservoir that includes four injection wells and nine production wells arranged in a five-point pattern. The model is modeled by 25 × 25 × 1 grid blocks with each grid length of 100 ft, each grid block is 20 ft thick, and the porosity of all grid blocks is 0.2. The physical properties of the reservoir are summarized in Table 6. The specific distribution of the modeled permeability is shown in Figure 4.

In this production optimization problem, the optimization variables consist of the injection rate for each injection well and the fluid recovery rate for the production well. The water injection rate ranges from 0 to 500 STB/DAY, while the water extraction rate for the production wells ranges from 0 to 200 STB/DAY. The thermal storage is utilized for a duration of 1800 days, and the decision time step is set at 360 days. Consequently, the dimensionality of the decision variable is 65.

The fitness function for this optimization problem is the NPV, which is determined by various factors. The oil price is set at 80.0 USD/STB, the cost of water injection is 5.0 USD/STB, and the cost of water treatment is also 5.0 USD/STB. To simplify the model, the average interest rate per annum is assumed to be 0%.

### 5.2. Analysis and Discussion of Experimental Results

Compare the optimization results of the model using CCWFO and the several classical evolutionary algorithms to showcase the effectiveness of the enhancements. These classical evolutionary algorithms include WFO, GWO, MFO, SMA, WOA, and PSO in Table 3. To ensure fairness in the experiment, each optimization was conducted five times, and the average of the last obtained NPV values was computed.

**Figure 5:**
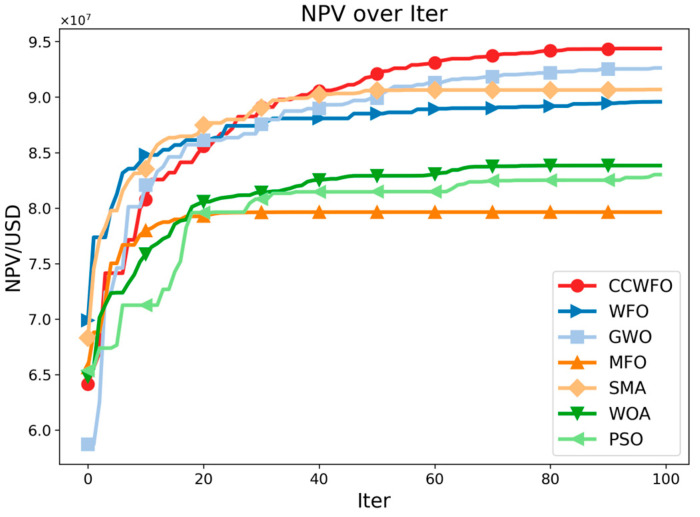
NPV obtained by the algorithms with iteration.

Figure 5 illustrates the optimal NPV values obtained using both methods as a function of the number of iterations. The red line represents CCWFO. From the figure, it is evident that CCWFO outperforms other algorithms significantly, consistently achieving higher NPV values within the same number of iterations.

**Figure 6:**
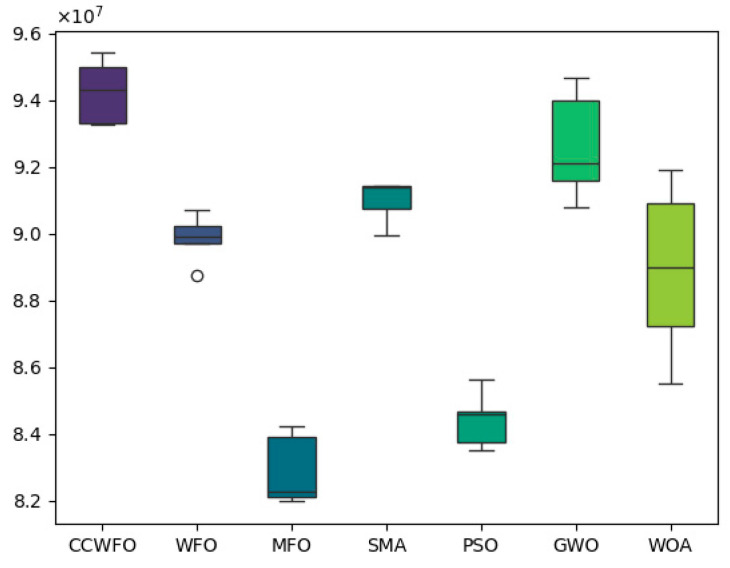
CCWFO vs. other algorithms: boxplot of best NPV values across 5 experiments.

**Figure 7:**
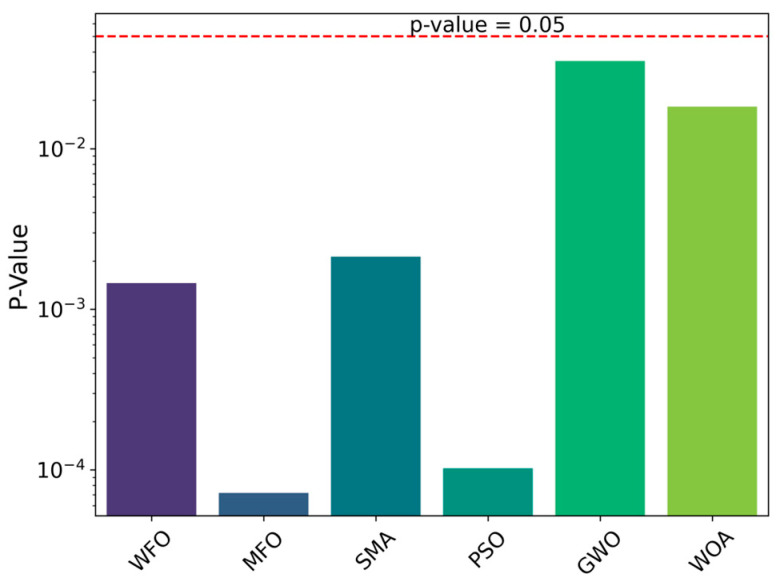
*p*-Value comparison between CCWFO and other algorithms.

Figure 6 shows the box plot comparison between CCWFO and other algorithms for obtaining optimal NPV in five experiments; it can be seen that in five experiments CCWFO obtains higher NPV as compared to other algorithms. From Figure 7, it can be seen that the *p*-values of the other traditional algorithms are less than 0.05, which proves that QCSCA has a significant advantage over the six other classical algorithms in terms of statistical significance.

**Figure 8:**
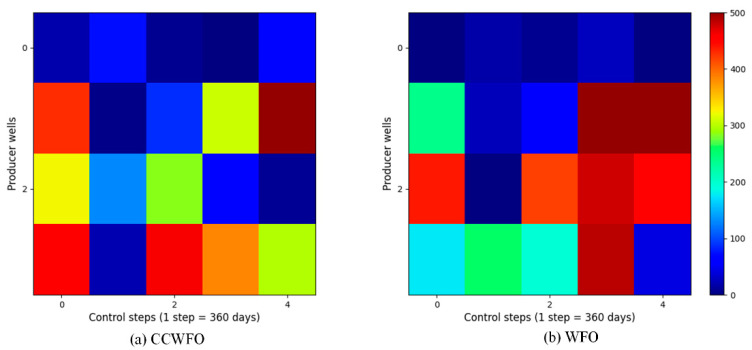
The optimal water-injection rate obtained by each algorithm for the three-channel model.

**Figure 9:**
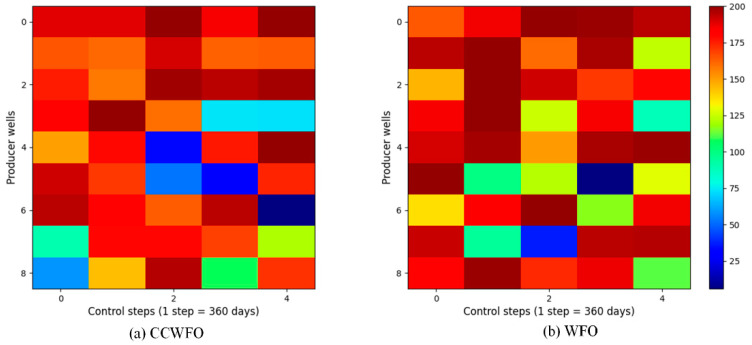
The optimal liquid-production rate obtained by each algorithm for the three-channel model.

For reasons of space, only the CCWFO and WFO well control schemes are given here; Figure 8 and Figure 9 illustrate the final optimization schemes of the water injection rate and liquid production rate for both CCWFO and WFO. The horizontal axis represents the practice step size, while the vertical axis represents the well number.

In Figure 8b, the regulation scheme for the injection wells obtained using WFO is displayed. It can be observed that the injection rates for the same wells in adjacent control step values vary significantly, resulting in an unstable scheme. This instability is not conducive to implementing the scheme in the field. Additionally, the fluctuating injection rates can cause excessive changes in bottomhole pressure, potentially damaging the reservoir and hindering sustainable development.

On the other hand, CCWFO, as shown in Figure 8a, yields a smoother production scheme compared to WFO. This smoother scheme is more favorable for implementation in the field, as it minimizes abrupt changes in injection rates and reduces the potential for negative impacts on the reservoir.

Overall, CCWFO demonstrates superior performance in generating more stable and smoother production schemes compared to WFO.

## 6. Conclusions

In this study, the implementation of the CCWFO optimizer is proposed by combining the CC mechanism with the WFO algorithm. The CC strategy enhances population diversity by promoting information exchange among individuals, resulting in improved global exploration capability. Comparative experiments conducted on benchmark functions on CEC2017 demonstrate that CCWFO consistently outperforms 10 other metaheuristic algorithms, yielding higher-quality solutions across different types of functions.

Furthermore, CCWFO is applied to solve the production optimization problem in reservoirs with a three-channel model, using a numerical model as an evaluator. The optimization results are compared with several classical evolutionary algorithms, and the experimental findings indicate that CCWFO achieves higher NPV within the same number of iterations. Additionally, CCWFO generates smoother production scenarios, which are more conducive to field development implementation.

In future research, we plan to explore and develop improved optimization methods. Additionally, we aim to closely integrate machine learning techniques with reservoir production scenarios to discover effective agent model strategies for solving complex large-scale production optimization problems.

## Figures and Tables

**Table 1:** Main parameters of the test experiment.

Parameter	Value
population size	30
problem dimension	30
number of runs	30
maximum number of evaluations	300,000

**Table 2:** CEC2017 benchmark functions.

Function	Function Name	Class	Optimum
F1	Shifted and Rotated Bent Cigar Function	Unimodal	100
F3	Shifted and Rotated Zakharov Function	Unimodal	300
F4	Shifted and Rotated Rosenbrock’s Function	Multimodal	400
F5	Shifted and Rotated Rastrigin’s Function	Multimodal	500
F6	Shifted and Rotated Expanded Scaffer’s F6 Function	Multimodal	600
F7	Shifted and Rotated Lunacek Bi-Rastrigin Function	Multimodal	700
F8	Shifted and Rotated Non-Continuous Rastrigin’s Function	Multimodal	800
F9	Shifted and Rotated Lévy Function	Multimodal	900
F10	Shifted and Rotated Schwefel’s Function	Multimodal	1000
F11	Hybrid Function 1 (N = 3)	Hybrid	1100
F12	Hybrid Function 2 (N = 3)	Hybrid	1200
F13	Hybrid Function 3 (N = 3)	Hybrid	1300
F14	Hybrid Function 4 (N = 4)	Hybrid	1400
F15	Hybrid Function 5 (N = 4)	Hybrid	1500
F16	Hybrid Function 6 (N = 4)	Hybrid	1600
F17	Hybrid Function 6 (N = 5)	Hybrid	1700
F18	Hybrid Function 6 (N = 5)	Hybrid	1800
F19	Hybrid Function 6 (N = 5)	Hybrid	1900
F20	Hybrid Function 6 (N = 6)	Hybrid	2000
F21	Composition Function 1 (N = 3)	Composition	2100
F22	Composition Function 2 (N = 3)	Composition	2200
F23	Composition Function 3 (N = 4)	Composition	2300
F24	Composition Function 4 (N = 4)	Composition	2400
F25	Composition Function 5 (N = 5)	Composition	2500
F26	Composition Function 6 (N = 5)	Composition	2600
F27	Composition Function 7 (N = 6)	Composition	2700
F28	Composition Function 8 (N = 6)	Composition	2800
F29	Composition Function 9 (N = 3)	Composition	2900
F30	Composition Function 10 (N = 3)	Composition	3000

**Table 3:** Hyperparameters for correlation algorithms.

Name	Parameters
CCWFO	$p_{l}\;$= 0.3; $p_{e}\;$= 0.7
WFO	$p_{l}\;$= 0.3; $p_{e}\;$= 0.7
SMA	/
WOA	$a_{1\;}$= [2, 0]; $a_{2}\;$= [−1, −2]; b = 1
PSO	$V_{max}$ = 6; $W_{max}$ = 0.9, $W_{min\;}$= 0.2; $C_{1\;}$= 2; $C_{2\;}$= 2
GWO	a = [2, 0]
MFO	b = 1; t = [−1, 1]; a = [−1, −2]
BMWOA	$a_{1}\;$= [2, 0]; $a_{2}\;$= [−1, −2]; b = 1
RCBA	$Q_{min\;}$= 0; $Q_{max}\;$= 2; r = 0.5
SCADE	scaling factor = [0.2, 0.8]; crossover probability = 0.8; a = 2
OBSCA	a = 2

**Table 4:** Experimental results of CCWFO and other algorithms on CEC2017.

	RANK	+/=/−	AVG
CCWFO	1	~	1.3793
WFO	2	14/11/4	1.8621
SMA	5	29/0/0	5.3793
WOA	9	29/0/0	7.7931
PSO	3	24/4/1	4.3793
GWO	4	29/0/0	4.8966
MFO	7	29/0/0	7.4138
BMWOA	8	29/0/0	7.4828
RCBA	6	27/2/0	7.2759
SCADE	11	29/0/0	9.4828
OBSCA	10	29/0/0	8.6552

**Table 5:** The *p*-values of CCWFO versus other algorithms on CEC2017.

	WFO	SMA	WOA	PSO	GWO
F1	1.73 × 10^−6^	1.73 × 10^−6^	1.73 × 10^−6^	3.39 × 10^−1^	1.73 × 10^−6^
F3	1.73 × 10^−6^	1.73 × 10^−6^	1.73 × 10^−6^	1.73 × 10^−6^	1.73 × 10^−6^
F4	4.07 × 10^−5^	1.73 × 10^−6^	1.73 × 10^−6^	8.61 × 10^−1^	1.73 × 10^−6^
F5	4.29 × 10^−6^	1.73 × 10^−6^	1.73 × 10^−6^	1.73 × 10^−6^	1.92 × 10^−6^
F6	1.73 × 10^−6^	1.73 × 10^−6^	1.73 × 10^−6^	1.73 × 10^−6^	1.73 × 10^−6^
F7	5.79 × 10^−5^	1.73 × 10^−6^	1.73 × 10^−6^	1.73 × 10^−6^	1.92 × 10^−6^
F8	1.49 × 10^−5^	1.73 × 10^−6^	1.73 × 10^−6^	1.73 × 10^−6^	3.18 × 10^−6^
F9	1.92 × 10^−6^	1.73 × 10^−6^	1.73 × 10^−6^	1.73 × 10^−6^	1.73 × 10^−6^
F10	6.88 × 10^−1^	1.73 × 10^−6^	1.73 × 10^−6^	1.73 × 10^−6^	3.52 × 10^−6^
F11	2.41 × 10^−4^	1.73 × 10^−6^	1.73 × 10^−6^	1.73 × 10^−6^	1.73 × 10^−6^
F12	8.92 × 10^−5^	1.73 × 10^−6^	1.73 × 10^−6^	3.32 × 10^−4^	1.73 × 10^−6^
F13	6.16 × 10^−4^	1.73 × 10^−6^	1.73 × 10^−6^	7.16 × 10^−4^	1.73 × 10^−6^
F14	5.04 × 10^−1^	1.73 × 10^−6^	1.73 × 10^−6^	1.73 × 10^−6^	1.73 × 10^−6^
F15	7.81 × 10^−1^	1.73 × 10^−6^	1.73 × 10^−6^	1.73 × 10^−6^	1.73 × 10^−6^
F16	6.42 × 10^−3^	1.73 × 10^−6^	1.73 × 10^−6^	1.73 × 10^−6^	2.22 × 10^−4^
F17	8.22 × 10^−3^	1.73 × 10^−6^	1.92 × 10^−6^	1.73 × 10^−6^	1.73 × 10^−6^
F18	7.04 × 10^−1^	1.73 × 10^−6^	1.73 × 10^−6^	1.73 × 10^−6^	1.73 × 10^−6^
F19	8.97 × 10^−2^	1.73 × 10^−6^	1.73 × 10^−6^	1.73 × 10^−6^	1.73 × 10^−6^
F20	2.13 × 10^−1^	1.73 × 10^−6^	1.73 × 10^−6^	1.73 × 10^−6^	1.92 × 10^−6^
F21	6.16 × 10^−4^	1.73 × 10^−6^	1.73 × 10^−6^	1.73 × 10^−6^	1.24 × 10^−5^
F22	6.64 × 10^−4^	8.19 × 10^−5^	1.73 × 10^−6^	1.82 × 10^−5^	2.37 × 10^−5^
F23	1.13 × 10^−5^	1.73 × 10^−6^	1.73 × 10^−6^	1.73 × 10^−6^	1.73 × 10^−6^
F24	2.88 × 10^−6^	1.73 × 10^−6^	1.73 × 10^−6^	1.73 × 10^−6^	2.05 × 10^−4^
F25	8.45 × 10^−1^	1.73 × 10^−6^	1.73 × 10^−6^	6.16 × 10^−4^	1.73 × 10^−6^
F26	1.11 × 10^−1^	1.13 × 10^−5^	1.73 × 10^−6^	8.69 × 10^−5^	2.22 × 10^−4^
F27	1.96 × 10^−2^	1.73 × 10^−6^	1.73 × 10^−6^	1.48 × 10^−2^	1.92 × 10^−6^
F28	1.95 × 10^−1^	1.73 × 10^−6^	1.73 × 10^−6^	8.73 × 10^−1^	1.73 × 10^−6^
F29	1.65 × 10^−1^	1.73 × 10^−6^	1.73 × 10^−6^	1.73 × 10^−6^	1.73 × 10^−6^
F30	5.58 × 10^−1^	1.73 × 10^−6^	1.73 × 10^−6^	9.75 × 10^−1^	1.73 × 10^−6^
	MFO	BMWOA	RCBA	SCADE	OBSCA
F1	1.73 × 10^−6^	1.73 × 10^−6^	2.60 × 10^−6^	1.73 × 10^−6^	1.73 × 10^−6^
F3	1.73 × 10^−6^	1.73 × 10^−6^	1.73 × 10^−6^	1.73 × 10^−6^	1.73 × 10^−6^
F4	1.73 × 10^−6^	1.73 × 10^−6^	1.15 × 10^−4^	1.73 × 10^−6^	1.73 × 10^−6^
F5	1.73 × 10^−6^	1.73 × 10^−6^	1.73 × 10^−6^	1.73 × 10^−6^	1.73 × 10^−6^
F6	1.73 × 10^−6^	1.73 × 10^−6^	1.73 × 10^−6^	1.73 × 10^−6^	1.73 × 10^−6^
F7	1.73 × 10^−6^	1.73 × 10^−6^	1.73 × 10^−6^	1.73 × 10^−6^	1.73 × 10^−6^
F8	1.73 × 10^−6^	1.73 × 10^−6^	1.73 × 10^−6^	1.73 × 10^−6^	1.73 × 10^−6^
F9	1.73 × 10^−6^	1.73 × 10^−6^	1.73 × 10^−6^	1.73 × 10^−6^	1.73 × 10^−6^
F10	1.73 × 10^−6^	1.73 × 10^−6^	1.73 × 10^−6^	1.73 × 10^−6^	1.73 × 10^−6^
F11	1.73 × 10^−6^	1.73 × 10^−6^	1.73 × 10^−6^	1.73 × 10^−6^	1.73 × 10^−6^
F12	1.73 × 10^−6^	1.73 × 10^−6^	1.73 × 10^−6^	1.73 × 10^−6^	1.73 × 10^−6^
F13	1.73 × 10^−6^	1.73 × 10^−6^	1.73 × 10^−6^	1.73 × 10^−6^	1.73 × 10^−6^
F14	1.73 × 10^−6^	1.73 × 10^−6^	1.73 × 10^−6^	1.73 × 10^−6^	1.73 × 10^−6^
F15	1.73 × 10^−6^	1.73 × 10^−6^	1.73 × 10^−6^	1.73 × 10^−6^	1.73 × 10^−6^
F16	1.73 × 10^−6^	1.73 × 10^−6^	1.73 × 10^−6^	1.73 × 10^−6^	1.73 × 10^−6^
F17	1.73 × 10^−6^	1.73 × 10^−6^	1.73 × 10^−6^	1.73 × 10^−6^	1.73 × 10^−6^
F18	1.73 × 10^−6^	1.73 × 10^−6^	1.73 × 10^−6^	1.73 × 10^−6^	1.73 × 10^−6^
F19	1.73 × 10^−6^	1.73 × 10^−6^	1.73 × 10^−6^	1.73 × 10^−6^	1.73 × 10^−6^
F20	1.92 × 10^−6^	1.73 × 10^−6^	1.73 × 10^−6^	1.73 × 10^−6^	1.73 × 10^−6^
F21	1.73 × 10^−6^	1.73 × 10^−6^	1.73 × 10^−6^	1.73 × 10^−6^	4.07 × 10^−5^
F22	1.73 × 10^−6^	5.75 × 10^−6^	1.73 × 10^−6^	2.13 × 10^−6^	2.35 × 10^−6^
F23	1.73 × 10^−6^	1.73 × 10^−6^	1.73 × 10^−6^	1.73 × 10^−6^	1.73 × 10^−6^
F24	1.73 × 10^−6^	1.73 × 10^−6^	1.73 × 10^−6^	1.73 × 10^−6^	1.73 × 10^−6^
F25	1.92 × 10^−6^	1.73 × 10^−6^	8.77 × 10^−1^	1.73 × 10^−6^	1.73 × 10^−6^
F26	1.73 × 10^−6^	3.88 × 10^−6^	3.18 × 10^−6^	1.73 × 10^−6^	1.73 × 10^−6^
F27	1.73 × 10^−6^	1.73 × 10^−6^	1.73 × 10^−6^	1.73 × 10^−6^	1.73 × 10^−6^
F28	1.73 × 10^−6^	1.73 × 10^−6^	7.19 × 10^−1^	1.73 × 10^−6^	1.73 × 10^−6^
F29	1.73 × 10^−6^	1.73 × 10^−6^	1.73 × 10^−6^	1.73 × 10^−6^	1.73 × 10^−6^
F30	1.73 × 10^−6^	1.73 × 10^−6^	1.73 × 10^−6^	1.73 × 10^−6^	1.73 × 10^−6^

**Table 6:** Properties of three-channel model.

Properties	Value
Reservoir grid	25 × 25 × 1
Depth	4800 ft
Initial pressure	4000 psi
Porosity	0.2
Compressibility	6.9 × 10^−5^ psi^−1^
Initial water saturation	0.2
Viscosity	2.2 cP

## Data Availability

The numerical and experimental data used to support the findings of this study are included within the article.
